# Study on the relationship between expression patterns of cocaine-and amphetamine regulated transcript and hormones secretion in porcine ovarian follicles

**DOI:** 10.1186/s40659-018-0154-y

**Published:** 2018-02-26

**Authors:** Pengfei Li, Jinzhu Meng, Jiongjie Jing, Qingling Hao, Zhiwei Zhu, Jianbo Yao, Lihua Lyu

**Affiliations:** 1 College of Life Science, Shanxi Agricultural University, Taigu, 030801 Shanxi China; 2 Wujiang College, Tongren University, Tongren, 554300 Guizhou China; 3 College of Animal Science and Technology, Shanxi Agricultural University, Taigu, 030801 Shanxi China; 4 Division of Animal and Nutritional Sciences, West Virginia University, Morgantown, WV 26506 USA

**Keywords:** Pig, CART, Estradiol, Progesterone, Granulosa cell

## Abstract

**Background:**

Cocaine-and amphetamine regulated transcript (CART) is an endogenous neuropeptide, which is widespread in animals, plays a key role in regulation of follicular atresia in cattle and sheep. Among animal ovaries, *CART* mRNA was firstly found in the cattle ovaries. CART was localized in the antral follicles oocytes, granulosa and cumulus cells by immunohistochemistry and in situ hybridization. Further research found that secretion of E_2_ was inhibited in granulosa cells with a certain dose of CART, the effect depends on the stage of cell differentiation, suggesting that CART could play a crucial role in regulating follicle atresia. The objective of this study was to characterize the CART expression model and hormones secretion in vivo and vitro in pig follicle granulosa cells, preliminarily studied whether CART have an effect on granulosa cells proliferation and hormones secretion in multiparous animals such as pigs.

**Methods:**

The expression levels of *CART* mRNA in granulosa cells of different follicles were analyzed using qRT-PCR technology. Immunohistochemistry technology was used to localize CART peptide. Granulosa cells were cultured in medium supplemented with different concentrations of CART and FSH for 168 h using Long-term culture system, and observed using a microscope. The concentration of Estradiol (E_2_) and progesterone (P) in follicular fluids of different test groups were detected by enzyme linked immunosorbent assay (ELISA).

**Results:**

Results showed that expression level of *CART* mRNA was highest in medium follicles, and significantly higher than that in large and small follicles (*P* < 0.05). Immunohistochemical results showed that CART were expressed both in granulosa cells and theca cells of large follicles, while CART were detected only in theca cells of medium and small follicles. After the granulosa cells were cultured for 168 h, and found that concentrations of E_2_ increase with concentrations of follicle-stimulating hormone (FSH) increase when the CART concentration was 0 μM. And the concentration of FSH reached 25 ng/mL, the concentration of E_2_ is greatest. It shows that the production of E_2_ needs induction of FSH in granulosa cells of pig ovarian follicles. With the increasing of CART concentrations (0.01, 0.1, 1 μM), E_2_ concentration has a declining trend, when the FSH concentrations were 25 and 50 ng/mL in the medium, respectively.

**Conclusions:**

These results suggested that CART plays a role to inhibit granulosa cells proliferation and E_2_ production, which induced by FSH in porcine ovarian follicular granulosa cells in vitro, but the inhibition effect is not significant. So we hypothesis CART maybe not a main local negative regulatory factor during porcine follicular development, which is different from the single fetal animals.

## Background

In mammals, ovarian follicular development is a continuous process during reproductive life span. Follicles develop through the primordial, primary, and secondary stage before acquiring an antral cavity, after which tertiary and preovulatory follicles successively form, and then oocytes are released after LH peak stimulation. In fact, only a few follicles undergo ovulation, most of the developing follicles will undergo atresia [1–4].

Ovarian follicles develop in a wave-like pattern in some species, such as pigs, human and cattle, each follicular wave is initiated by a transient elevation of FSH, then a cohort of follicles begin to grow, among which some follicles growing fast transform into the dominant follicles, while the rest become the subordinate follicles that will undergo atresia [5–9].

Cocaine-and amphetamine regulated transcript (CART), discovered initially by Douglass et al. via differential display RT-PCR analysis of brains of rats administered cocaine [10], is expressed mainly in central nervous system or neuronal origin cells [11], which is involved in a wide range of behaviors, such as regulation of food intake, energy homeostasis, and reproduction [12–15]. CART, as a potent anorectic peptide in hypothalamus, is regulated by leptin [16]. Since leptin stimulation of GnRH release can be blocked by CART protein antibody in vitro, the action of leptin on reproductive neuroendocrine axis may be mediated by CART [17]. As specific CART receptors have not been identified, the regulatory mechanisms of CART biological activities are still unknown.

Follicular growth and development is a complex process, which is not only precisely regulated by some endocrine factors, but also some locally produced intraovarian factors [3, 18–20]. A previous study reported that *CART* mRNA and CART peptide are expressed in bovine oocyte, and ovarian cells such as cumulus cells, and granulosa cell layer of antral follicles but not preantral follicles, suggesting a potential role of CART in the atresia of antral follicles [21]. Based on these findings, we hypothesized that CART may also play a role as a potential local regulator in the process of porcine follicular development. To investigate the relationship between CART and pig follicular development, we determined CART mRNA expression level in porcine follicles of different sizes and the concentrations of E_2_ and P in follicular fluid of those follicles, the localization of CART peptide was also detected by immunohistochemistry technology, and explored the effects of CART on granulosa cells proliferation and E_2_ secretion by in vitro culture. Our results indicated that CART may be involved in the process of porcine antral follicle development, yet its role may not be mediated by regulating the concentration of E_2_ and P.

## Methods

### Animal care

All animal procedures were performed with strict accordance with the recommendations in the Guide for the Care and Use of Laboratory Animals of the National Institutes of Health.

### Follicles and granulosa cells collection

Ten ovaries were collected from five female Large White pigs at the local slaughterhouse (Taigu, Shanxi, China). Follicles of 2–8 mm were dissected free from ovarian stroma and washed in 70% alcohol and DPBS solution for a few seconds. The follicles were classified into large (diameter > 5 mm), medium (3 mm < diameter < 5 mm) and small (diameter < 3 mm) groups, and placed in culture medium. Follicular fluid from each group follicles was aspirated and frozen on dry ice and stored at − 20 °C until hormone detection. Follicles were cut in half, and granulosa cells were collected from follicle internal wall and frozen in liquid nitrogen and stored at − 80 °C until RNA isolation.

### RNA isolation and cDNA synthesis

Total RNA was isolated using Trizol (Takara, Dalian, China) according to the manufacturer’s instructions. Isolated RNA was dissolved in 30 µL of RNase free water. Before cDNA synthesis, 2 µL of total RNA were mixed with 2 µL of 5 × gDNA Eraser Buffer, 1 μL of gDNA Eraser (Takara, Dalian, China) and 5 μL of RNase free water and incubated at 42 °C for 2 min to remove genomic DNA. 0.8 μg RNA was mixed with 4 μL of 5 × PrimeScript^®^ Buffer 2, 1 μL of RT Primer Mix, 1 μL of PrimeScript^®^ RT Enzyme Mix I and 4 μL of RNase Free water (Takara, Dalian, China), and then incubated at 37 °C for 15 min followed by incubation at 85 °C for 5 s to synthesize cDNA, which was stored at − 20 °C until use.

### Quantitative real-time PCR (qRT-PCR)

The relative expression level of *CART* mRNA in pig follicles of different sizes was measured by qRT-PCR. qRT-PCR was performed using 20 µL reaction volume containing 10 µL of SYBR^®^ Green *premix Ex Taq*™II, 0.4 µL of ROX Reference Dye II(Takara, Dalian, China), 0.8 µL of forward and reverse primer, respectively, 2 µL of cDNA and 6 µL nuclease free water. Reactions were run on a 7500 Real Time PCR system (Thermo Scientific, Beijing, China) for 45 cycles at 95 °C for 15 s followed by 60 °C for 1 min. *β*-*Actin* gene was used as the endogenous control. Primers were designed using Primer 3 (http://primer3.ut.ee/), porcine *CART* primer was designed according to *Sus scrofa CART* mRNA, the primers are listed in Table 1. The relative mRNA expression level of *CART* was calculated using the comparative 2^−ΔΔCT^ method [22]. The CART standard curve had a slope of − 3.124 (Eff. = 109.0%). The *β*-*Actin* standard curve had a slope of − 3.166 (Eff. = 106.9%).

**Table 1:** Primer sequences used in this study

Primer name	Sequence (5′to 3′)	Tm (°C)	Size (bp)
*CART*-F	TATGTGTGACGCAGGAGAGC	59.3	102
*CART*-R	AAGGAATTGCAGGAGGTTCC	59.8	
*β*-*Actin*-F	CCAGCACCATGAAGATCAAG	60.0	93
*β*-*Actin*-R	ACATCTGCTGGAAGGTGGAC	60.0	

*F* sense primers, *R* antisense primers

### Immunohistochemical localization of CART

Samples of adult ovary stroma were collected at a local abattoir from ovaries of three different animals. Samples were placed in a plastic tissue cassette, fixed with 4% (vol/vol) paraformaldehyde solution, and embedded in paraffin. Immunohistochemical localization of the CART peptide was performed using previously described procedures [23]. Rabbit anti-rat CART (55–102) polyclonal antisera (Phoenix Pharmaceuticals, Inc., Belmont, CA) (1:1000 dilution) was used in the analysis. Parallel controls were used, including sections incubated with a similar dilution of normal rabbit serum or rabbit anti-CART serum that had been pre-incubated overnight at 4 °C with 10 μg/mL rat CART (55–102) peptide (American Peptide Co., Sunnyvale, CA). Four serial sections from each sample were examined.

### Granulosa cells cultured in vitro

Long-term culture system was performed using our previously procedures [24, 25]. Granulosa cells were cultured in a humidified environment of 5% CO_2_ and air at 37 °C for 168 h, medium was replaced with fresh medium every 48 h. Granulosa cells were harvested after termination of culture, washed by DPBS and digested using tryptase, and cell numbers were determined [26]. The situation of cells were observed and collected images after cultured for 48, 96, 144 and 168 h. At the end of culture, 340 μL medium were pooled from 2 adjacent wells per treatment, stored at − 20 °C for measurement of hormone.

### Estradiol and progesterone enzyme-linked immunosorbent assay (ELISA)

Concentrations of E_2_ and P in follicular fluid of follicles at different sizes were detected using pig free estradiol and progesterone ELISA kit (Blue gene, Shanghai, China) according to the manufacturer’s instructions. The ELISA plates were read with a microplate reader (Thermo Scientific, Shanghai, China) to record the optical densities and the concentration of E_2_ and P derived from standard curve. The assay sensitivity was set as 0.5 pg/mL. The inter- and intra-assay CVs for E_2_ were 8.3 and 7.6%, respectively, and the inter- and intra-assay CVs for P were 9.2 and 5.8%, respectively.

The determination method of E_2_ concentration in medium was same to that in follicular fluid, standard curve was drew, concentration of each group was calculated。

### Statistical analysis

Three biological reduplicates were used in all experiments mentioned above. The expression level of *CART* mRNA in granulosa cells of porcine follicles at different sizes and concentrations of E_2_ and P in follicular fluid were analyzed by one way ANOVA using SPSS computer software (IBM, USA). E_2_ concentration in culture medium was determined by Duncan-method of multiple comparisons. Data were presented as mean ± SE.

## Results

### Detection of *CART* mRNA in granulosa cells of porcine follicles

RT-PCR detection of *CART* mRNA and *β*-*Actin* gene in granulosa cells of porcine follicles at different sizes were shown through agarose gel electrophoresis. The products of *β*-*Actin* gene amplification in large, medium and small follicles were all 93 bp in size. *CART* mRNA amplification products in large, medium and small follicles were 102 bp in size.

The nucleotide sequence of the porcine *CART* and *β*-*Actin* cDNAs derived from RNA of follicular granulosa cells was 102 and 93 bp, respectively (data not shown). The nucleotide sequence of porcine CART shared 100% homology with that of *Sus scrofa* CART.

### *CART* mRNA expression levels in large, medium and small follicles

**Fig. 1:**
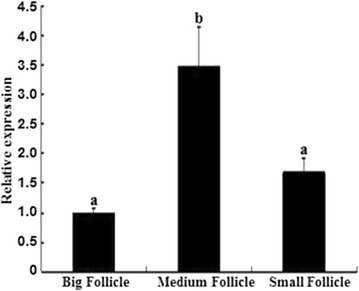
Quantitative real-time PCR analysis of *CART* mRNA expression in big, medium and small follicles. Superscript small letters indicate significantly different at the level of 0.05, same letters mean no significantly difference while different letters mean significantly difference. (n = 3 each; least square mean ± SEM)

Quantitative real time PCR analysis revealed that the expression level of *CART* mRNA was significantly higher in medium follicles than that in large and small follicles (*P* < 0.05) (Fig. 1).

### Concentrations of E_2_ and P in follicular fluid of large, medium and small follicles

**Fig. 2:**
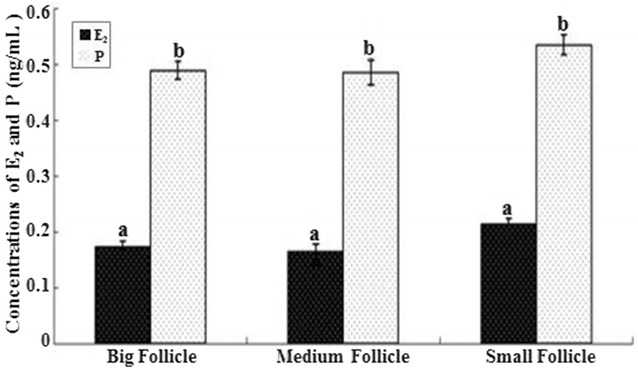
Estradiol and progesterone concentrations in big, medium and small follicles. Superscript small letters indicate significantly different at the level of 0.05, same letters mean no significantly difference while different letters mean significantly difference. (n = 3 each; least square mean ± SEM)

The concentrations of E_2_ and P in follicular fluid of large, medium and small follicles are showed in Fig. 2. No significant differences in the concentrations of each hormone (E_2_ and P) were observed in follicles of different sizes.

### Intraovarian expression of CART protein

**Fig. 3:**
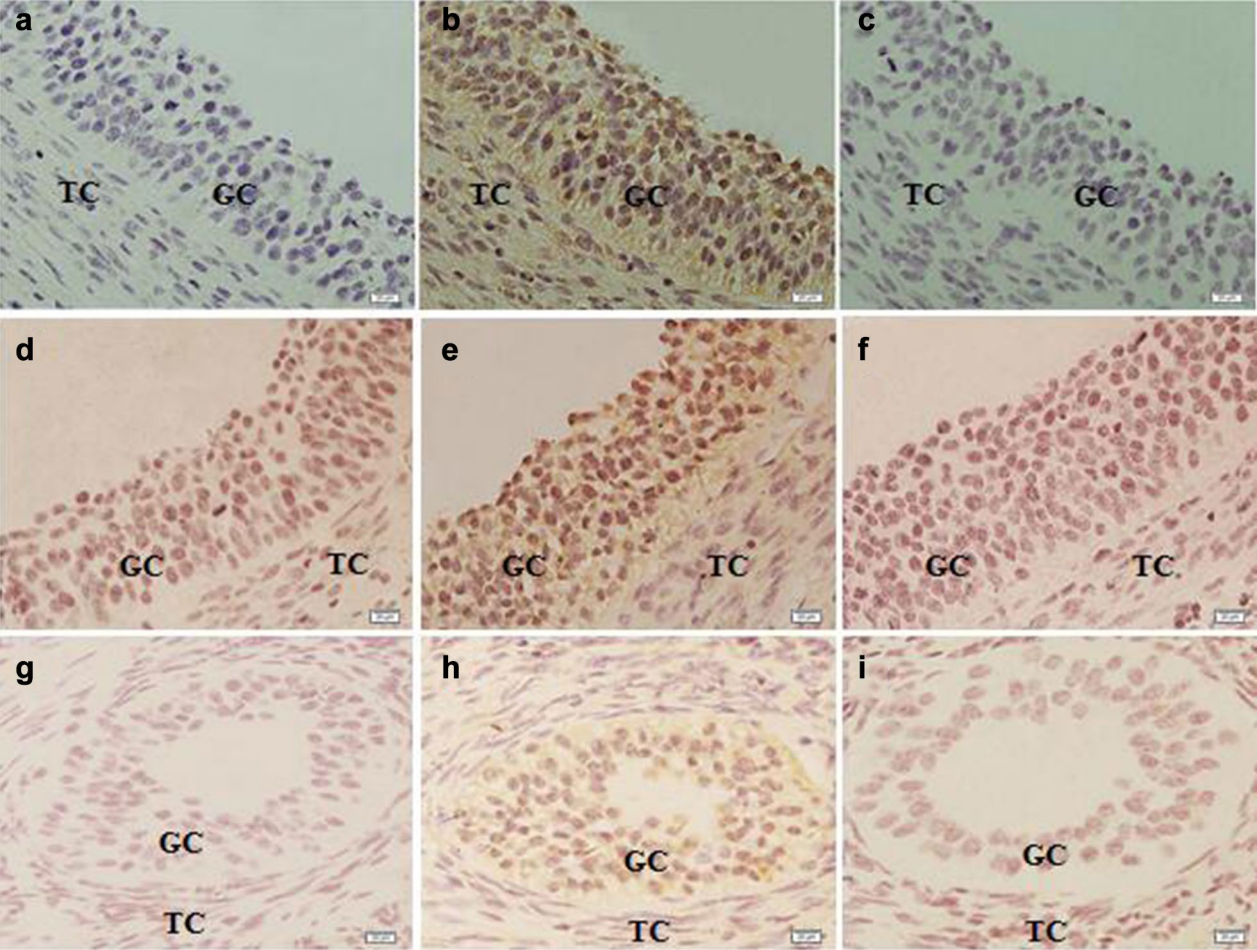
Immunohistochemical localization of CART peptide within the porcine ovary. **a**–**c**, Adjacent section micrograph of big follicle; **d**–**f**, adjacent section micrograph of medium follicle; **g**–**i**, adjacent section micrograph of small follicle. **a**, **d**, **g**, Pre-immune serum; **b**, **e**, **h**, rabbit anti-rat CART; **c**, **f**, **i**, pre-absorbed rabbit anti-rat CART. GC, granulosa cell layer; TC, thecal cell layer; **a**–**f**: Magnification, ×400; scale bar, 20 µM

The intra-ovarian localization of CART peptide was determined by immunohistochemistry (Fig. 3). CART immunoreactivity was localized to the granulosa cells of big, medium and small follicles (Fig. 3b, e, h). Significant immunoreactivity in the granulosa cells was not detected when adjacent sections were incubated with normal rabbit serum (Fig. 3a, d, g) or when the CART antiserum was pre-absorbed with excess CART peptide (Fig. 3c, f, i).

### Response doses effect of CART on E_2_ production and granulosa cells proliferation under long-term culture with FSH treatment

**Fig. 4:**
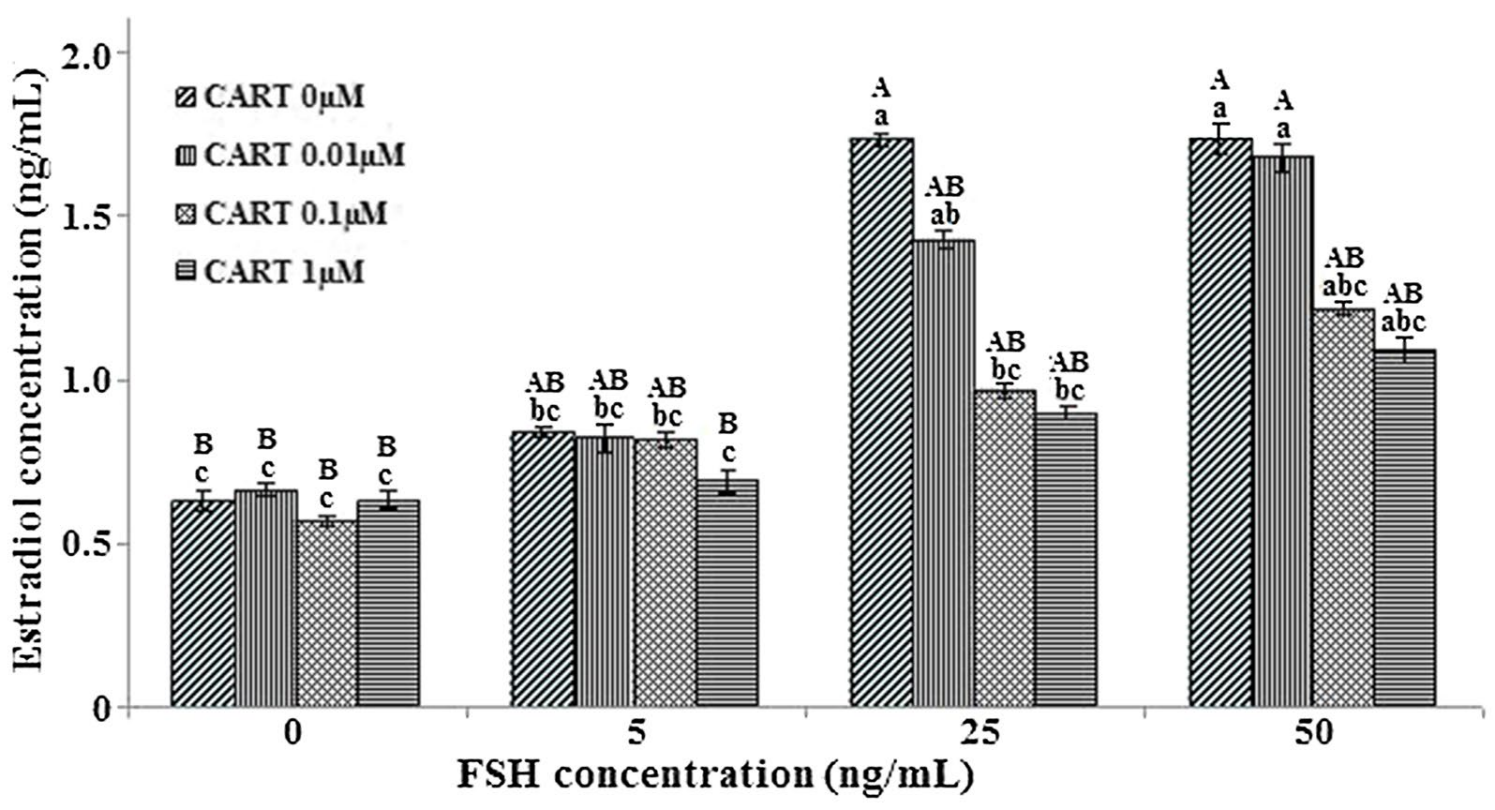
Effects of CART on FSH induced estradiol production of pig follicular granulosa cells after 168 h in vitro culture. Superscript small letters and capital letters indicate significantly different at the level of 0.05 and 0.01, Values with the same letters were not significantly different and values with the different letters were significantly different at the level of 0.01 or 0.05. (n = 3 each; least square mean ± SEM)

Comparing with control group, with the concentrations of FSH were increasing (5, 25, 50 ng/mL) in medium, the concentrations of E_2_ are on the rise, when the CART concentration was 0 μM. And the concentration of FSH reached 25 ng/mL, the secretion of E_2_ is greatest. It shows that the production of E_2_ needs induction of FSH in granulosa cells of pig ovarian follicles. With the increasing of CART concentrations (0.01, 0.1, 1 μM), E_2_ concentration has a declining trend, when the FSH concentrations were 25 and 50 ng/mL in the medium, respectively, but the generation of E_2_ is significantly suppressed when pretreatment with 25 ng/mL FSH, 0.1 and 1 μM CART (*P* < 0.05) (Fig. 4).

**Fig. 5:**
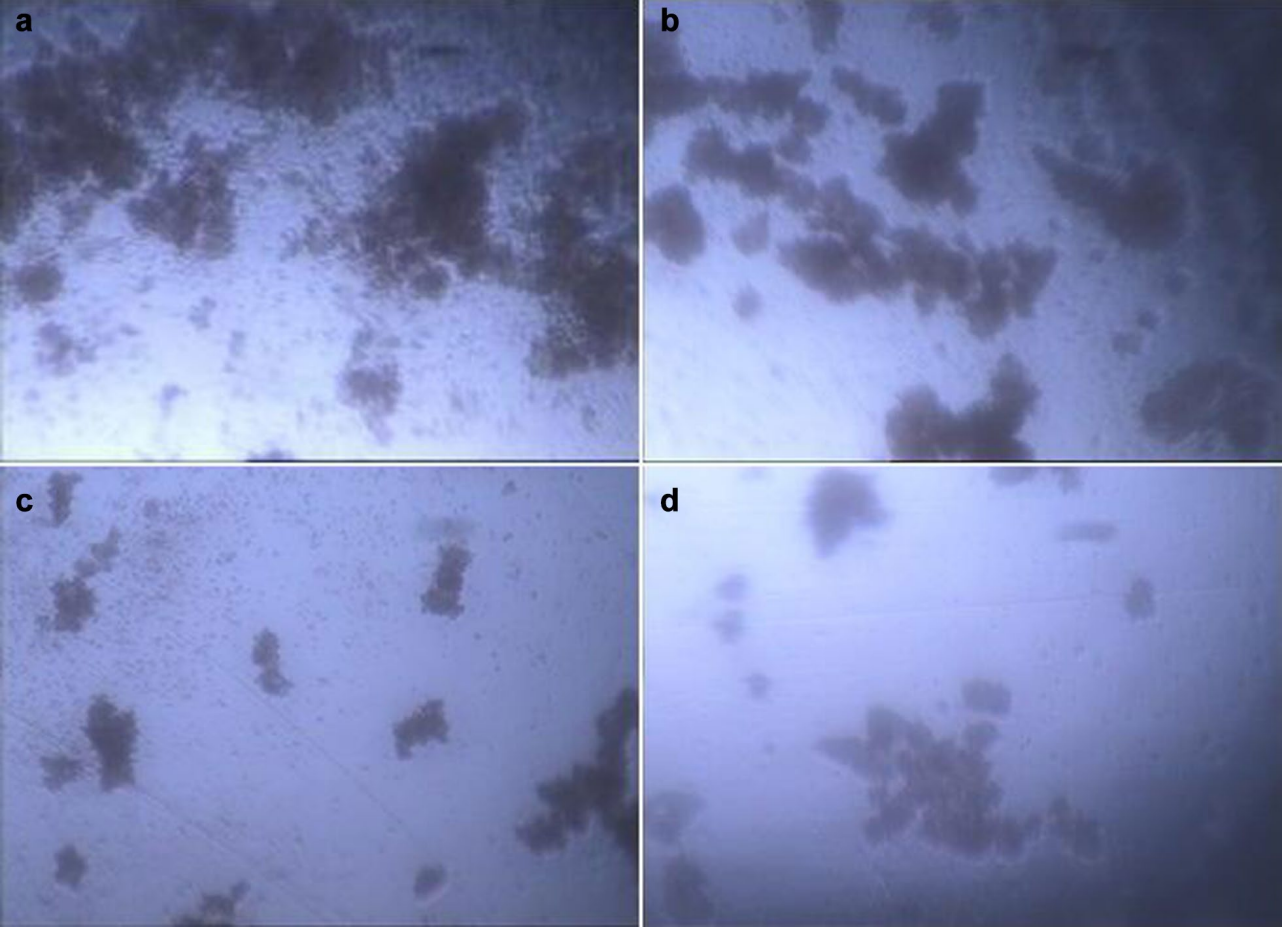
The micrograph of granulosa cells after 168 h in vitro culture with FSH (25 ng/mL) in culture system, **a** CART 0 µM; **b** CART 0.01 µM; **c** CART 0.1 µM; **d** CART 1 µM. Magnification, ×100

The images of granulosa cells in vitro culture shows that clusters of granulosa cells were obviously decrease with the concentration of CART increase at FSH (25 ng/mL) in culture system (Fig. 5). This is consistent with the detection results of E_2_ (Fig. 4), indicating that decrease of granulosa cells numbers lead to the decrease of E_2_ secretion.

## Discussion

The process of follicle growth and development is regulated by various endocrine factors and intra-ovarian factors. As our understanding of the well-established endocrine regulation of antral follicle growth and development increasing, attention in recent years has been focused on both identification and subsequent contribution of locally produced regulatory molecules that are involved in antral follicle growth and developmental regulation. Results of the present study demonstrated that the previously described anorectic neuropeptide CART had a higher expression level in the granulosa cells of medium follicles than that of big and small follicles. CART expression was potentially associated with follicle development. However, other results of the present study indicated that the concentrations of E_2_ and P in follicular fluid of large, medium and small follicles had no significant difference. Results support potential local regulatory role for CART in porcine follicular development without regulating the production of E_2_ and P.

Our results indicate that E_2_ secretion decrease with the increase of CART under same FSH concentration, suggested that CART might plays an suppressive role on the proliferation of pig ovarian follicle granulosa cells. Previous research found CART can restrain E_2_ secretion of bovine follicular granulosa cells in vitro, and plays a negative regulatory role during follicular development [27, 28]. Our study confirmed CART can inhibit the proliferation of pig ovarian follicle granulosa cells, meanwhile CART could promote granulosa cell apoptosis of porcine ovarian follicles [29]. But inhibition effect of CART is not significant.

Being classified as a somatostatin-like peptide, the amino acid sequence of CART peptide was first reported for sheep in hypothalamus [30]. Subsequently more and more researches were focused on the identity of CART. Until 1995 when Douglass and his co-workers [10] identified CART as a mRNA species whose expression increased acutely after psychomotor stimulant administration. The nucleotide and predicted amino acid sequences of CART from rat [10], human [31], mouse [32], sheep [33] and cattle [34] have been reported previously and were highly homologous among different species. However, the expression of *CART* mRNA in the porcine ovary has not been reported.

Previous studies have reported that CART was expressed in many tissues, including pituitary gland, adrenal gland [11, 35, 36], stomach [36], and intestines [36, 37]. However, reports about CART on mammalian gonads are rare. Murphy et al. [36, 38] did not detect immunoreactive CART peptide in rat ovaries and testis. Within gonadal tissues, CART expression has been reported in the goldfish ovary [38] and in nerves that innervate the epididymis of rat testis [39]. However, the present study detected *CART* mRNA and peptide expression in granulosa cells of porcine antral follicles. Smith, et al. [12] also found that both *CART* mRNA and protein were expressed in oocyte, cumulus cells, and granulosa cells of antral follicles in bovine ovary.

Our study demonstrated that the *CART* mRNA and peptide expressed in granulosa cells of porcine antral follicles at various development stages and the medium follicles had the highest expression level, suggesting that CART may play an important role in antral follicle development by regulating differentiation of granulosa cells. The exact mechanisms about how CART exerts its regulatory role in granulosa cells need to be further explored.

It is acknowledged that study design was not optimal due to follicles (large, medium, small) were used in CART mRNA expression and hormone determination. For the demarcation of follicles in different stages of multiparous animals, unlike single fetal animals whose follicles can be clearly divided into dominant and subordinate follicles. Despite such limitations, our results indicates that the concentrations of E_2_ and P in follicular fluid of large, medium and small follicles had no significant difference, which suggests that CART has no direct regulatory effect on the production of these two hormones in porcine antral follicles. Follicular growth and development are regulated by endocrine factors [40, 41], including E_2_ and P, which are important indicators to reflect the state of follicular growth. Smith et al. [12] demonstrated that CART had an inhibitory effect on in vitro production of E_2_ by granulosa cells.

## Conclusion

CART leads to decrease of E_2_ production by inhibiting granulosa cells proliferation, which induced by FSH in porcine ovarian follicular granulosa cells. We hypothesis CART maybe not a main local negative regulatory factor during porcine follicular development, which is different from the single fetal animals.
